# Mortality Associated with Orofacial Clefts in Brazil

**DOI:** 10.3390/dj13070282

**Published:** 2025-06-23

**Authors:** Amanda de Andrade Costa, Hildeth Maisa Torres Farias, Daniella Reis B. Martelli, Verônica Oliveira Dias, Brazilian Oral Cleft Group, Ricardo D. Coletta, Hercílio Martelli Junior

**Affiliations:** 1 Primary Care/Health Sciences Postgraduate Program, State University of Montes Claros Claros (UNIMONTES), Montes Claros 39401-089, MG, Brazil; amanda.deandrade@saude.mg.gov.br (A.d.A.C.); daniela.martelli@unimontes.br (D.R.B.M.); veronica.dias@unimontes.br (V.O.D.); 2 Epidemiological Surveillance and Occupational Health Center, Regional Health Superintendency of Montes Claros, Montes Claros 39408-111, MG, Brazil; hildeth.farias@saude.mg.gov.br; 3 Department of Oral Diagnosis and Graduate Program in Oral Biology, School of Dentistry, University of Campinas, Piracicaba 13414-018, SP, Brazil; coletta@fop.unicamp.br; 4 Oral Pathology and Oral Medicine, School of Dentistry, State University of Montes Claros (UNIMONTES), Montes Claros 39400-000, MG, Brazil

**Keywords:** unified health system, mortality, orofacial cleft

## Abstract

**Background/Objectives:** Orofacial clefts are congenital anomalies that cause substantial morbidity and mortality. This study aimed to investigate temporal and geographic trends in mortality among Brazilian individuals with orofacial clefts listed as the underlying cause of death on death certificates. **Methods:** A retrospective cross-sectional study was conducted using data from the Department of Informatics of the Brazilian Unified Health System (DATASUS) from 1996 to 2023. **Results:** The mortality information system registered 987 deaths related to orofacial clefts, with 880 patients under 1 year of age. There was a downward trend in annual mortality rates from 1996 to 2019, followed by an increase from 2020 to 2023. The main associated cause of death was respiratory and cardiovascular disorders. The mortality rate for infants under 1 year with orofacial clefts showed greater variation than did the mortality rate of children who died of other causes. The reduction in mortality rates from 1996 to 2019 occurred during the expansion and strengthening of DATASUS and its coordination with other levels of healthcare. The rise in mortality between 2020 and 2023 coincided with a reduction in surgical procedures due to the COVID-19 pandemic. **Conclusions:** This study revealed a decline in deaths from orofacial clefts in Brazil over several decades. These findings emphasize the importance of addressing preventable causes of death, including respiratory infections and malnutrition. High mortality within the first year of life—particularly among newborns under 28 days—highlights a critical shortage of pediatricians and its impact on care for individuals with craniofacial anomalies.

## 1. Introduction

Orofacial clefts are congenital anomalies that result from failures in the embryonic development of the face and/or palate. These include cleft lip and palate (CLP), cleft lip only (CLO), and cleft palate only (CPO) [1]. The estimated global prevalence of cleft lip and/or palate is 1 in 700 live births, while in Brazil, approximately 1 in 650 live births are affected by this malformation. The prevalence varies according to sex, ethnicity, and geographic region [2,3]. 

This anomaly has a multifactorial etiology that is influenced by genetic, epigenetic, and environmental factors, such as maternal exposure to smoking, alcohol consumption, and certain medications during pregnancy [4]. Studies have shown that the presence of orofacial clefts increases the risk of mortality compared with the general population [5,6,7,8]. 

The mortality rate among children with orofacial clefts is higher than that of children without clefts during the first 2 years of life, and the average life expectancy is significantly lower for children with clefts [9,10]. Factors associated with an increased risk of mortality in multivariable regression models include lower birth weight, the presence of other congenital or chromosomal anomalies, and a reduced number of prenatal consultations [9]. Additionally, orofacial clefts may be part of a broader spectrum of congenital anomalies or a syndrome associated with an elevated risk of mortality [10].

Surveillance of birth and mortality related to orofacial clefts is essential for healthcare planning [11]. In many cases, orofacial clefts are not lethal on their own, and with the expansion of CLP treatment centers, more individuals with these congenital defects are surviving into adulthood [6,8,12]. Furthermore, United Nations member states, including Brazil, are committed to achieving the Sustainable Development Goals, which include reducing neonatal and child mortality from preventable causes and non-communicable diseases through prevention and treatment—categories that include children with common congenital defects such as orofacial clefts [13]. 

This study aimed to investigate temporal and geographic trends in mortality among Brazilian individuals with orofacial clefts listed as the underlying cause of death on death certificates. In addition, we sought to identify the demographic characteristics and contributing causes most commonly associated with these deaths.

Although studies have explored the epidemiology of orofacial clefts in Brazil [14], few have evaluated longitudinal mortality trends using national-level data. Understanding how mortality varies over time and across regions is crucial for tailoring public health interventions. Moreover, despite the availability of treatment through the SUS, barriers to accessing care persist—particularly in Northern and Northeastern regions, which often lack specialized surgical centers [14,15].

The central research question guiding this study is “What are the temporal, demographic, and geographic patterns of mortality associated with orofacial clefts in Brazil, and what factors contribute to the persistence of mortality, particularly among infants under one year of age?”

By addressing this question, this study seeks to generate evidence to inform targeted public health strategies aimed at reducing preventable deaths, particularly in the most vulnerable regions and age groups. Furthermore, it contributes to strengthening national policies for the comprehensive care of individuals with craniofacial anomalies.

## 2. Materials and Methods

A retrospective, population-based cross-sectional study was conducted using birth and mortality records from the Department of Informatics of the Brazilian Unified Health System (DATASUS) (https://datasus.saude.gov.br/informacoes-de-saude-tabnet/ accessed on 22 March 2024) [16]. Because of the evolution of data management processes and information technology resources, DATASUS has expanded the scope of information collected over the years. As a result, the availability of data used in this research has varied start dates.

The analysis was based on data with national geographical coverage, including regions and federative units of Brazil. For analyses requiring birth information, live birth declarations that recorded infants with orofacial clefts from 2010 to 2023 were used, along with general birth records from 1996 to 2023. Although the Live Birth Information System (SINASC) and the adoption of the Live Birth Certificate as the standard model for use throughout Brazil were implemented in 1990, it was only in 2000 that Brazil adopted a new version of the certificate, which included a field to record the presence or absence of “congenital malformation and/or chromosomal anomaly”. In 2010, an updated version was introduced, expanding the section on congenital anomalies by adding a space to describe all congenital anomalies observed in the newborn. The study period was defined to begin in 2010, marking the implementation of this updated certificate, and to end in 2023, the most recent year with data free from inconsistencies such as duplications and coding errors at the time of collection. However, 1996 was also set as a starting point for the study because it marks the adoption of the International Classification of Diseases, Tenth Revision (ICD-10) in Brazil. Data from 2024 were excluded because the public database had not yet been finalized and remained subject to updates, including the removal of duplicates, correction of data entry errors, and resolution of data transfer failures. Mortality analyses were based on the underlying cause of death, as recorded on DCs for individuals of all age groups using ICD-10 codes Q35, Q36, and Q37 [17], which refer to CPO, CLO, and CLP, respectively, covering the period from 1996 to 2023.

To identify other causes contributing to the deaths of patients with orofacial clefts, records of multiple causes of death from 2006 to 2023 were also included. Multiple causes, also referred to as contributing causes, indicate other diagnoses related to the death. The DC is divided into Part I and Part II. Part I is further subdivided into lines (a, b, c, d), which must be filled out from bottom to top in a logical sequence, with the underlying cause of death recorded on line d and multiple causes on lines a, b, and c. In Part II of the DC, other contributing causes of death that were not part of the causal chain listed in Part I are recorded [18].

All causes recorded on lines a, b, and c of Part I, as well as those listed in Part II of the DC, were identified as multiple causes. Understanding the multiple causes of death in patients with orofacial clefts supports the implementation of preventive care, continuous monitoring, and personalized treatments, helping to reduce the risk of fatal complications and improve the quality of life for this population. Mortality records from DATASUS provided information on skin color, age group, location and region of death, sex, education level, and marital status of all individuals with orofacial clefts who died during the study period.

Information was organized by year. To calculate the infant mortality rate due to orofacial clefts per 1000 live births, the total number of deaths of children under 1 year of age with orofacial clefts and the total number of live births from 2010 to 2023 were used. To calculate the overall mortality rate due to orofacial clefts, the total number of deaths among individuals with orofacial clefts and the total number of general deaths occurring in the same period (1996–2023) were considered. To analyze trends in mortality due to orofacial clefts, joinpoint regression analysis was applied using the Joinpoint Regression Program software, Version 5.2.0 (National Cancer Institute, Bethesda, MD, USA). This model calculates the annual percentage change in the mortality rate. For this study, the dependent variable was the mortality rate due to orofacial clefts (per 1000 inhabitants), and the independent variable was the year.

As a retrospective, population-based study based on secondary data, this analysis may be subject to underreporting, misclassification, and variations in diagnostic coding over time. Additionally, death certificates may fail to capture all contributing anomalies or comorbidities. Despite these limitations, the breadth and consistency of the DATASUS database allow for robust trend analysis over time.

This study used secondary data available in the DATASUS database from the Ministry of Health. Because the data did not include subject identification, this study was exempt from ethics committee review.

## 3. Results

**Figure 1:**
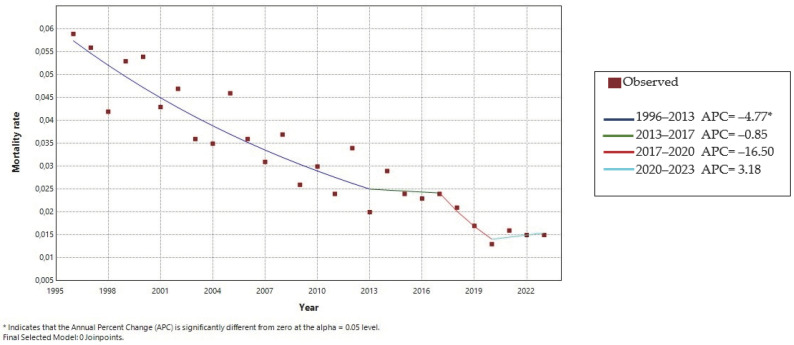
Mortality trend of patients with orofacial clefts, 1996–2023.

Between 1996 and 2023, the mortality information system of the SUS registered 987 deaths of individuals with orofacial clefts as the underlying cause. Of these, 419 were due to CPO, 355 to CLP, and 213 to CLO. Deaths were predominantly recorded in the southeast region (*n* = 333), followed by the northeast (*n* = 288), south (*n* = 170), north (*n* = 114), and midwest (*n* = 82). Figure 1 illustrates the distribution of deaths among patients with orofacial clefts over the 28 years analyzed. The mortality rate reached its highest value of 20.27 in 2012 and its lowest of 9.65 in 2019, demonstrating a significant downward trend. From 1996 to 2013, the annual percentage change in mortality was −4.77% (*p* = 0.000002; 95% confidence interval: −6.1 to −3.4). Between 2013 and 2017, the decline continued but at a slower pace, suggesting a stabilization in the mortality rate. Subsequently, from 2017 to 2020, a renewed decrease in mortality was observed. However, from 2020 to 2023, the trend reversed, with an increase in the mortality rate among individuals with orofacial clefts.

The profile of the deaths was characterized by infants under 1 year of age (*n* = 880 deaths), male sex (*n* = 533), and Caucasian individuals (*n* = 402). The majority of deaths occurred in a hospital setting (*n* = 809). Among deaths in infants under 1 year of age, 477 (48.3%) took place within the first 28 days of life (Table 1). Results regarding the type of orofacial clefts by sex indicate that for CLO (*n* = 130), CPO (*n* = 223), and CLP (*n* = 180), mortality was more prevalent in males. 

**Figure 2:**
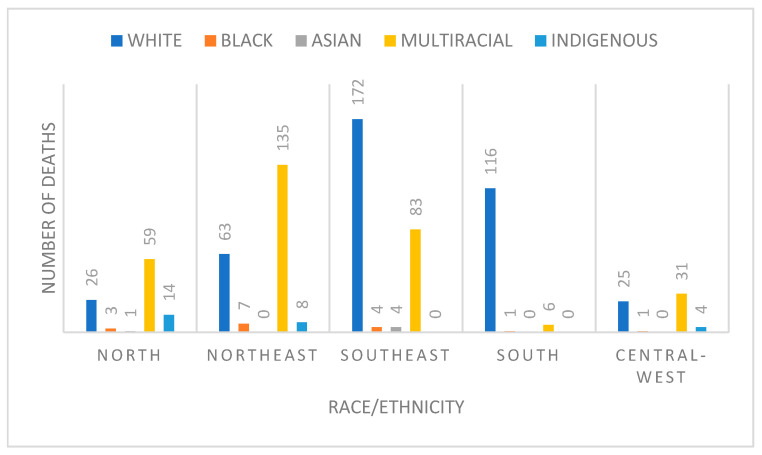
Number of deaths of individuals under 1 year of age with orofacial clefts by region of Brazil and race/ethnicity. DATASUS, 1996–2023.

Examining the distribution of deaths in infants under 1 year of age with orofacial clefts, it was observed that reported skin color varied by region of residence in Brazil, with a predominance of deaths among Caucasian infants in the southeast and multiracial infants in the northeast (Figure 2).

**Figure 3:**
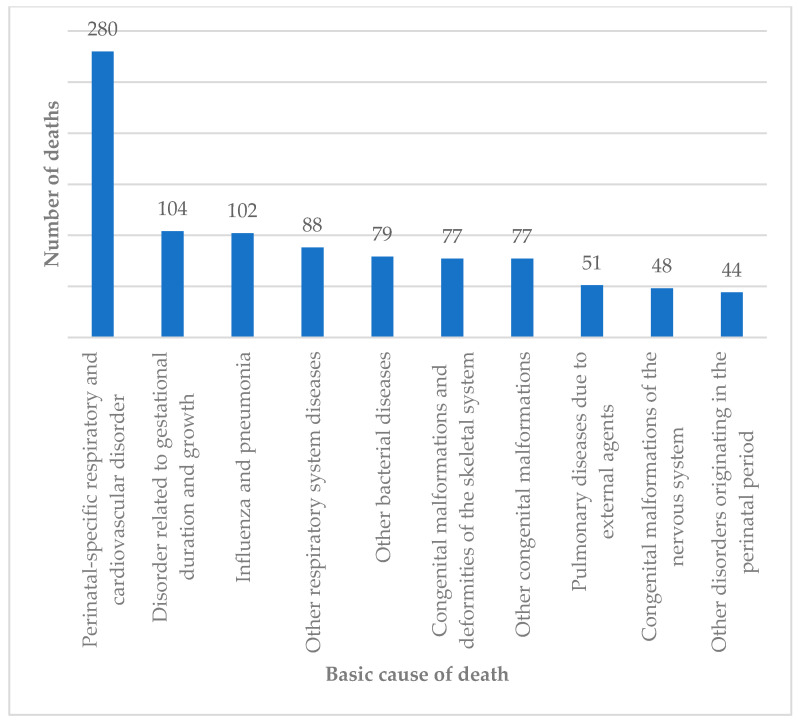
Number of deaths of individuals with orofacial clefts by multiple causes, according to ICD-10 chapters. DATASUS, 2006–2023.

The causes of death among individuals with orofacial clefts varied widely, but data available for the 17-year period (2006–2023) showed that in addition to the 664 individuals whose orofacial clefts contributed to death, respiratory and cardiovascular disorders (*n* = 280) were the most common associated causes, particularly among infants under 1 year of age. Other causes of death reported on the DC included disorders related to gestational duration and growth; influenza and pneumonia; bacterial diseases; congenital malformations of the nervous system, eyes, ears, face, and heart; congenital skeletal malformations; and pulmonary diseases induced by external agents (Figure 3).

**Figure 4:**
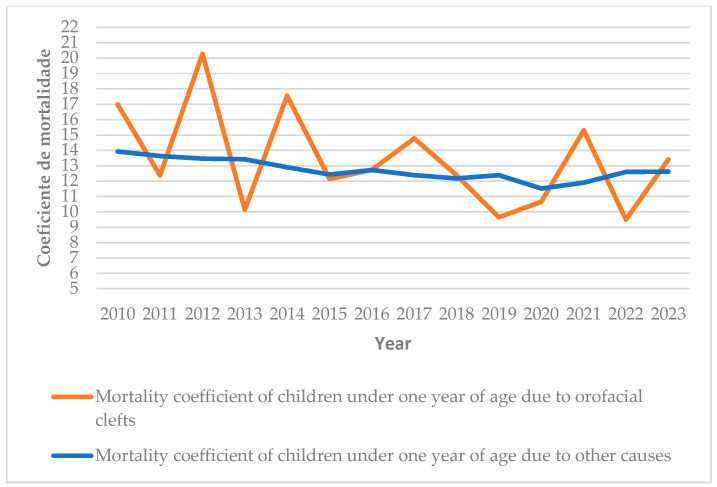
Mortality rate of children under 1 year of age who died of orofacial clefts and other causes. DATASUS, 2010–2023.

When calculating the mortality coefficient for infants under 1 year of age with orofacial clefts from 2010 to 2023 and comparing it with the mortality coefficient for children who died of other causes during the same period, the data in Figure 4 show that mortality among infants with orofacial clefts was higher. Although there was variability over the years studied, a downward trend was observed in the later years of the study. In the years with peaks in infant mortality due to orofacial clefts (2012, 2014, and 2021), the mortality coefficient for children who died of other causes did not show significant fluctuations and displayed a stable trend (Figure 4).

## 4. Discussion

The risk of mortality among individuals with orofacial clefts is higher in developing than developed countries because of socioeconomic disparities, limited access to healthcare, inadequate service infrastructure, and a higher prevalence of infectious diseases [8,19,20]. Mortality rates vary globally, with 20.3 deaths per 1000 live births with oral clefts reported in the United States, 36/1000 in England, 31.8/1000 in the United Kingdom, and 12.2/1000 in South Korea [7,11,21,22].

Geographic analysis of death records in the present study revealed regional disparities, with the southeast region exhibiting the highest number of deaths. This may be attributed to the region’s higher population density, which creates greater demand for healthcare services—potentially exposing systemic deficiencies—or to a more comprehensive recording of orofacial cleft-related deaths. Despite receiving the largest allocation of financial resources for orofacial cleft care (USD 22,896,078.83 [60.3%]) [14], the southeast’s population growth underscores the need for ongoing investment reviews and expansions to ensure efficient resource management and strategic planning. Furthermore, the geographic concentration of orofacial cleft treatment centers near higher education institutions—predominantly located in the southeast—likely contributes to the observed population density. The mortality rates observed in other regions highlight the urgent need for public health policies tailored to the specific needs of each area [14], ensuring equitable access to care and addressing regional disparities in the management of orofacial clefts.

Strategies should be considered to reduce mortality associated with orofacial clefts, particularly in underserved regions of Brazil. Establishing regional referral networks and deploying mobile surgical units may help address the limited geographic availability of specialized treatment centers. These mobile teams could deliver timely surgical interventions and postoperative care in remote areas lacking adequate infrastructure [23]. Telehealth initiatives for prenatal and pediatric counseling can facilitate early diagnosis, referral, and ongoing clinical monitoring of affected infants, especially in regions without resident specialists [24]. National guidelines could better define mandatory referral timelines and age-based care parameters to avoid delays in treatment. National guidelines could further define mandatory referral timelines and age-based care benchmarks to prevent delays in treatment [25,26]. These findings also highlight the need to reevaluate current public funding policies. Promoting equitable resource allocation, including incentives to train and retain pediatricians and craniofacial specialists in underserved areas, could help address structural disparities in service delivery [14]. These strategies offer a roadmap for public health interventions and policy improvements aimed at preventing avoidable deaths and enhancing the quality of life for individuals born with orofacial clefts.

The findings from the present study are consistent with previous research conducted in South America, which included a consecutive series of in-hospital live-born infants with any form of isolated or associated typical cleft lip and/or palate in 47 hospitals across Argentina, Brazil, Bolivia, Chile, Colombia, Ecuador, and Venezuela. The multicentric study demonstrated that systematic pediatric care—delivered through weekly pediatric visits and parental education between the 7th and 28th days of life—did not significantly reduce neonatal mortality but did decrease neonatal hospitalization days by an average of six days among infants with associated cleft lip and/or palate [27]. These results underscore the importance of structured pediatric follow-up during the neonatal period, particularly for infants with complex health needs. Implementing similar systematic care programs in Brazil could be a cost-effective public health strategy to reduce the substantial financial and healthcare burdens associated with orofacial clefts, especially for families residing in underserved areas. 

In this study, most deaths occurred among Caucasian individuals. However, a study conducted in the United States identified a higher number of deaths among Black, Hispanic, and Native American children, with Black individuals with orofacial clefts having a two-fold higher likelihood of death than Caucasians [11]. Despite this, there is limited information regarding the mortality of individuals with orofacial clefts in relation to race. The existing literature on sex aligns with the results presented here, showing higher mortality in males [21,28,29].

In Brazil, care and treatment for orofacial clefts are provided free of charge by SUS in specialized services authorized by the Ministry of Health, with private services acting in a complementary role. The reduction in the mortality rate from 1996 to 2013 can be attributed to several factors, including the expansion and strengthening of SUS, established by law in 1990 [30] (https://www.planalto.gov.br/ccivil_03/leis/l8080.htm accessed on 22 March 2024). This improved access to universal and equitable medical care for the Brazilian population, including diagnosis and treatment for specialized conditions such as orofacial clefts. Another contributing factor to the reduction in deaths was the creation of the Family Health Program in 1994 and its integration with other levels of healthcare, allowing for increased access to care with continuous monitoring, education, and awareness, multidisciplinary support, and improved early detection and treatment. In 1995, nutrition and breastfeeding programs, as well as vaccination campaigns, were developed. These may have contributed to reduced mortality through the prevention of infections and child malnutrition [28,30]. Social and educational support programs also improved families’ ability to seek early treatment and provide better care for children with orofacial clefts [31]. In 1997, partnerships between the government and the American non-governmental organization Operation Smile provided free surgeries, professional training, and multidisciplinary support for orofacial cleft care [32].

Until 1993, Brazil had only two facilities providing assistance to individuals with orofacial clefts, both located in the state of São Paulo. From that point onward, several new services were established throughout the country, with 27 units offering cleft treatment by 2013—likely contributing to the persistent reduction in mortality over the years studied. By 2025, there were 34 cleft treatment units authorized by SUS across Brazil, equipped with the necessary physical infrastructure, equipment, and human resources to provide clinical, surgical, and rehabilitation services for these individuals. However, 12 Brazilian states still do not have authorized treatment centers, with 64.7% of existing facilities concentrated in the southeast and south regions and only two services located in the north (Tocantins and Pará) [15] (https://cnes2.datasus.gov.br/Mod_Ind_Habilitacoes_Listar.asp?VTipo=0401&VListar=1&VEstado=00&VMun=&VComp=&VContador=34&VTitulo=H).

## 5. Conclusions

This study provides evidence that after 24 years of declining mortality, there was an increase in the mortality rate for orofacial clefts between 2020 and 2023. This rise coincided with the coronavirus disease 2019 pandemic, during which the World Health Organization recommended a reduction in surgical procedures—a factor that may have impacted the rehabilitation, monitoring, and mortality of individuals with orofacial clefts in the country. The temporal association between the increase in mortality since 2020 and the COVID-19 pandemic suggests a causal link, but other factors, such as inconsistencies in reporting, regional disparities in access to care, and delays in treatment, may also have influenced the increase in numbers. The number of children under 1 year of age who died of causes related to orofacial clefts was higher than the mortality rate for children who died of other causes in the same age group. The prevalence of mortality in the first year of life, particularly among neonates under 28 days old, highlights the shortage of pediatric specialists as a public health concern and a critical gap in care for children with orofacial clefts. Deaths due to respiratory infections and malnutrition further underscore the need for targeted interventions to reduce preventable mortality in this vulnerable population.

## Figures and Tables

**Table 1:** Characterization of deaths according to age, sex, and race, 1996–2023.

Age	*N*
0–27 days	477
28–364 days	403
1–4 years	68
5–9 years	8
10–14 years	6
15–19 years	3
25–29 years	2
30–34 years	4
40–44 years	1
45–49 years	1
50–54 years	4
65–69 years	1
70–74 years	3
75–79 years	2
≥80 years	3
Unknown	1
Sex	*n*
Male	533
Female	446
Race	*n*
Caucasian	402
Multiracial	314
Unknown	224
Indigenous	26
Black	16
Asian	5

## Data Availability

The original data presented in this study are openly available at https://datasus.saude.gov.br/informacoes-de-saude-tabnet/. Accessed on 22 March 2024.

## Abbreviations

The following abbreviations are used in this manuscript:

SUS	Unified Health System
CLP	Cleft lip and palate
CLO	Cleft lip only
CPO	Cleft palate only
DATASUS	Department of Informatics of the Brazilian Unified Health System
SINASC	Live Birth Information System
ICD-10	International Classification of Diseases, Tenth Revision
DC	Death certificate
